# The Health of a Migrant Population: A Phenomenological Study of the Experience of Refugees and Asylum Seekers in a Multicultural Context

**DOI:** 10.3390/nursrep14020104

**Published:** 2024-05-31

**Authors:** Paola Arcadi, Mariachiara Figura, Silvio Simeone, Gianluca Pucciarelli, Ercole Vellone, Rosaria Alvaro

**Affiliations:** 1 Department of Biomedicine and Prevention, University of Rome Tor Vergata, 00133 Rome, Italy; paola.arcadi@students.uniroma2.eu (P.A.); mariachiara.figura@students.uniroma2.eu (M.F.); ercole.vellone@uniroma2.it (E.V.); rosaria.alvaro@uniroma2.it (R.A.); 2 Department of Experimental and Clinic Medicine, University of Catanzaro Magna Grecia, 88100 Catanzaro, Italy; silvio.simeone@unicz.it; 3 Department of Nursing and Obstetrics, Wroclaw Medical University, 50-367 Wroclaw, Poland

**Keywords:** refugees, migration, cultural competence, health needs, qualitative research, community care

## Abstract

Refugees and asylum seekers bring with them a plurality of cultures, traditions, and values that could prove crucial in influencing perceived health needs, requests for intervention, or willingness to undergo specific health treatments. Although studies have focused on the health consequences of forced migration, in recent years, there has been a lack of information on how refugees and asylum seekers represent their experiences of perceived health needs and elements that influence well-being, in a community context. This study aims to explore the experience of refugees and asylum seekers in an Italian multicultural community about perceived health needs. A qualitative phenomenological study was conducted with an interpretive approach. The data were collected using a semi-structured face-to-face interview. The interviews were transcribed, read thoroughly, and analyzed. Nineteen refugees and asylum seekers were interviewed. Three main themes were extracted: (1) the centrality of the family to feel healthy; (2) feeling part of a community; and (3) stability and security. The results confirm that health needs, experiences, and different cultural representations of health and illness should be read and addressed with a culturally competent vision. This study was not registered.

## 1. Introduction

Migration is a global, significant, complex, and fast-growing phenomenon influenced by geographic, socio-economic, and political factors [1]. In the last decade, 100 million people have fled their homes to seek refuge within or outside their countries’ borders [2]. According to the Mid-Year Trend report, there was exponential growth in migration in 2022. More than 84 million people worldwide were forced to migrate due to violence, insecurity, or the effects of climate emergency [2]. Europe has experienced an unprecedented influx of refugees and asylum seekers (ASs); about 1.5 million people have arrived since 2015, more than 1 million of whom have sought asylum after fleeing countries affected by war, conflict, or economic crisis [3].

Refugees and ASs differ from other migrants because they did not choose to migrate but were forced to flee their countries [2]. The migration phase following flight is often unpredictable, exhausting, and sometimes marked by encounters with different traumatic experiences. The refugees’ experience is punctuated by conditions of uncertainty in which the person is “suspended” between two different societies and forms of culture. Indeed, those who flee their country cross the geographic, cultural, and psychological borders of the hosting countries [4].

The social, economic, political, and cultural consequences generated by hundreds of thousands of forced migrants within the European continent also manifest themselves in Italy, a country that has seen a significant increase in landings in the last few years. In 2019, Italy reported about 354,700 refugees, including AS, representing 5.7 percent of the total migrants [5]. It is estimated that in 2015–2020 about half a million migrants arrived on Italian shores, crossing the central Mediterranean route that connects the two shores of the Mediterranean. Inevitably, this phenomenon led the hosting society to confront the challenge of reception and inclusion [6,7].

On the health front, refugees and AS, arriving from the Mediterranean often, report trauma from the physical and psychological violence experienced during the migration [8,9,10]. Furthermore, poverty, social isolation, armed conflict, human rights violations, stressors related to forced migration, and separation from family make it difficult to maintain optimal health status [11,12].

The so-called “healthy migrant effect” is known in the literature, which recognizes better health outcomes in migrants than those of the native population [12]. Nonetheless, post-migration exposure to difficult environmental and cultural situations, such as unsafe or unhealthy living and working conditions, changes in lifestyles related to the acculturation process, and limited access to health services [11] can be the cause of a reduction in their health capital. All of these challenges are amplified by language and cultural barriers [13] and the non-uncommon perception by migrants that their needs, values, beliefs, and cultural practices are not considered.

All these aspects could complicate the process of adaptation and social integration within host countries and lead to health inequalities [8,13]. Therefore, recalling the principles guiding transcultural nursing, to protect the refugees’ health, international and national policies should insist on their integration into social and health services, as outlined in the Sustainable Development Goals of Agenda 2030 [14] on promoting social and labor insertion, fostering cultural assimilation of the other, and inclusion in health services. Indeed, as described by the transcultural model, nurses should emphasize the importance of knowledge about health problems affecting specific cultural groups, ascertaining cultural substrates, and individual and contextual needs to develop a culturally congruent plan of care [15]. Furthermore, migrants bring with them a plurality of cultures, traditions, and values that could be crucial in influencing perceived health needs, requests for intervention, or willingness to undergo specific treatments [16].

A large body of literature indicates that the exposure of refugees and ASs to traumatic war-related events has a significant impact on the persistence of mental health disorders [17,18,19]. Refugees and ASs share a past trauma that threatens their mental integrity. As described in the literature, indeed, trauma is one of the most significant risk factors for depression [17], post-traumatic stress disorder [18], and anxiety [19]. Many studies have led to greater interest in post-migration issues, especially stressors and how these affect refugees’ psychological well-being [9,10,11], while others were focused on analyzing specific indicators of social disadvantage, such as unemployment, poor living conditions, single status, and limited social networks [20].

However, although studies have focused on the health consequences of forced migration in recent years, there is still a lack of information on how refugees and ASs represent their experiences of perceived health needs and elements influencing health and well-being (Luiking et al., 2019). Refugees and ASs vitally contribute to the debate about their treatment and health, but the dialogue surrounding them seems limited to those in positions of institutional power [21]. Based on the critical assumption that, as of today, we are in a context of pluralism and heterogeneity that cries out to be heard and understood, for health care to meet the health needs of migrant populations adequately, it should be adapted to their needs, in terms of their self-perceived wants and needs [13]. A study that explores the subjective dimension of the health of refugees and ASs would provide important information on guiding the interventions to take charge of these populations in a proactive manner.

The study aims to explore the experiences of refugees and ASs in Italy concerning perceived health needs and the elements that influence health and well-being.

## 2. Materials and Methods

### 2.1. Design

A qualitative phenomenological study with an interpretive approach was conducted. Interpretative phenomenological analysis (I.P.A.) focuses on the in-depth meaning of the participants’ experiences [22]. On the one hand, it adopted an idiographic approach because of the individual case investigation. On the other hand, it also used an interpretive one, following the principles of Husserlian hermeneutic phenomenology [23]. Finally, the phenomenological perspective was used to obtain knowledge from phenomena, free from theoretical presuppositions that assign meanings to experience a priori [24]. Researchers run into the personal representations of the experiences of people immersed in a linguistic, relational, cultural, and physical world, taking part in intersubjective meaning-making, where one cannot avoid interpretation but must reflect on one’s role in producing such interpretations.

### 2.2. Sampling and Recruitment

The study was conducted in Camini, a rural village in southern Italy located above the coast of the Ionian Sea, where a project for the reception and integration of refugees and ASs is active and which represents an example of coexistence between native and immigrant populations [25,26].

A propositional criterion was used for the sampling. Since the standard duration of participation in a reception project for each migrant is six months, participants were recruited who had been included in the Camini reception project for at least three months. This minimum period included people who could consciously tell about their experience concerning the subjects being studied. A shorter experience in the project probably would not have been sufficient to provide comprehensive information on health needs perceived and accrued over time. Migrants with diagnosed mental health disorders were excluded. Involvement in the study lasted until data saturation was reached. No recruited person refused to participate in the study.

The sample comprised nineteen people, equally distributed gender, and an average age of 30.6 years (SD 8.7). Participants came from nine countries and arrived in Italy between 2014 and 2021. Three of them were Nigerian (15.79%), four were Moroccan (21.05%), four were Syrian (21.05%), two were Somali (10.53%), one was Lebanese (5.26%), two were Pakistani (10.53%), one was from Bangladesh (5.26%), one was from Senegal (5.26%), and one was from Ghana (5.26%). The main sociodemographic results are reported in Table 1.

### 2.3. Data Collection

The data was collected in December 2022 in Camini at an agreed-upon location with the participants. After identifying the possible study participants, the host project managers individually contacted them to agree on the date and time of the interview. Previously, the participants had not had contact with the researchers.

Following the chosen methodology, each researcher involved in the study performed bracketing before data collection, writing down ideas, preconceptions, and beliefs about the phenomenon under investigation. This first step is crucial because researchers’ preconceived notions could influence data analysis in studies using a phenomenological interpretive approach [24]. By performing this “reflective technique” before data collection and analysis, researchers can be more careful to avoid introducing biases that could negatively influence research. Data were collected using a semi-structured face-to-face interview [22]. This type of interview was chosen because it is particularly informative, allowing the researcher to create the framework for the topics covered. However, the respondent’s responses determine how interviews are conducted. Furthermore, the semi-structured interview guide provides a clear set of instructions for the interviewers and, at the same time, can provide reliable and comparable qualitative data [22].

The interviewers invited each participant to answer a description of their health experience starting with an open and general question, such as: “At this time in your life, what do you need most to be well?”. Then, the interviews took the form of free interview conversations, using open-ended reflections and response request to facilitate discussion. The interviewers probed participants’ experiences of health needs, trying to highlight their feelings, changes, challenges, and expectations.

During the interviews, no one was present other than the researchers and cultural mediators for migrants. Interviews were conducted in the researchers’ language (Italian), and cultural mediators intervened in cases where questions were not understood by migrants, by interpreting Arabic, French, and Pidgin English languages. During the interviews, the researchers maintained an empathetic attitude, expressing warmth and reassurance, to facilitate the participants narrating their experiences [22]. Field notes were written, helpful in recording personal reflections, notes relating to the setting, and the non-verbal language used by the interviewees. According to Corbin and Strauss [27], interviews were conducted without interruption until participants said they had nothing more to add or until new information emerged. Data saturation was reached after 19 interviews. A sociodemographic questionnaire explicitly created for this project was used to collect information on the characteristics of the participants.

The established qualitative research standards criteria for reporting were followed in writing the report [28].

### 2.4. Data Analysis

The average duration of the interviews was 25 min. The interviews were audio-recorded and transcribed in full by assigning an identification code to each interview. The translation of the interview parts into the native language was performed by a cultural mediator unrelated to the study to keep the text’s original meaning as much as possible and to control the cultural bias in the data analysis phase [29]. The interviews and field notes were independently read and reread in depth by two interviewers (PA and SS), initially annotating the descriptive, linguistic, and conceptual elements that emerged from the text [22].

Subsequently, the emerging themes were identified, organized in a table to implement a comparison (grouping of themes), and finally grouped within superordinate themes. Each superordinate theme was linked to the underlying themes, which, in turn, were related to the participants’ original quotes [22]. A consensus validation was performed between the two researchers. No discrepancies or disagreements emerged. Finally, the themes identified were exemplified with a descriptive narration and illustrated with participant quotations.

### 2.5. Rigour

The criteria of credibility, transferability, and reliability, described by Lincoln and Guba [30], were considered to ensure the study’s methodological rigor. Member checking was performed. The findings were shared with participants, who were asked to confirm the emerging themes and share any additional information. At this point, translation was performed to compile the research report. The processes of translating and back-translating the in-text citations to enhance the themes were carried out and focused on conceptual content rather than literal equivalents. The purpose of the translation was to look for the conceptual equivalent of a word or phrase and not a word-for-word translation. Thus, it was ensured that the original meaning of the data obtained was respected.

### 2.6. Ethical Considerations

The procedures applied in this study followed the principles outlined in the Declaration of Helsinki [31]. Approval was obtained from the University Ethics Committee (protocol registration number 160.21). The purpose of the study was explained to each participant, and written informed consent was acquired to ensure anonymity, confidentiality, and data protection. All participants were assured that they could withdraw from the study at any time. Each interview was assigned a sequential alphanumeric code, with no possibility of identifying participants.

## 3. Results

The data analysis identified three main themes: (1) the centrality of the family to feel healthy; (2) feeling part of a community; and (3) stability and security. Each theme encompasses different sub-themes, summarized in Table 2.

### 3.1. Centrality of the Family to Feel Healthy

In this theme, the family system, a microsystem with a pivotal role within the individual’s life, represents the place of affection and stable and deep relationships and can enhance the individual’s health and quality of life. The family is configured as the primary organism that acts as the glue between its various constituent members and in which the individual can feel safe, experience relationships with others and recognize themselves, and carries with it a value system that, rooted in traditions, is projected into future generations.

“My family is the meaning of my life, and I carry with me what I have been taught to give to my children even if we are far away. This makes me feel good”

(KP11).

The importance of the affective dimension with family members translates into a supportive relational network, and the unity of the members can be a source of strength in facing all kinds of obstacles, as well as being the essential element in creating a positive family environment.

“When we do something, we all decide it together. It is the strength we have in this new world”

(JQ10).

Three specific sub-themes generate the theme of family centrality.

#### 3.1.1. The Symbolic Role of Parental Function

The first sub-theme delves into the symbolic meaning of parenthood in the context of the man–woman relationship. Children are seen as the key positive outcome of this union. The importance placed on parenting translates into a compelling push for individuals to cultivate specific caregiving skills tailored to the needs of their children. Chief among these skills is the effort to ensure a prosperous future for one’s descendants. This motivation often underlies migration decisions, as exemplified by the quote: “It is always for our children (that we left the country), both for me and for my husband, we do and are doing everything for our children” (FU06). On the contrary, the inability to adequately respond to the needs of one’s children generates feelings of discomfort, which manifest themselves in tangible physical discomfort, as expressed in the quote: “Being sick is when my children have needs, but I can’t satisfy them “ (MN13).

#### 3.1.2. The Reunification of the Family Unit

The second sub-theme focuses on the imperative of family reunification, recognized as a vital mechanism for promoting family cohesion and well-being. In many cases, migrants face the complex and emotionally taxing experience of family separation. Although they jointly agreed to the migratory journey out of an imperative to ensure stability for themselves and their relatives, they are grappling with the high cost of involuntary removal. They recognize that efforts to sustain connections through long-distance communication fail to alleviate the discomfort of detachment. This poignant reality precipitates feelings of nostalgia that have a significant impact on their well-being, as articulated in the lament, “It’s impossible to feel good because I haven’t seen my mother for 5 years and my father for 20 years”.

#### 3.1.3. Individual Well-Being Depends on the Well-Being of the Family

The third, and final, sub-theme concerns the close relationship between individual well-being and family well-being. Interviewees underline that their health is closely linked to the general well-being of their family unit. They attest: «To feel good I need the main things, that is, that the family is well» (LO12). Conversely, they recognize that they experience discomfort when a family member, such as a spouse, becomes ill, as evidenced by the sentiment “if the husband is ill” (MN13). This feeling often catalyzes a proactive process aimed at improving the well-being of family members, as illustrated by this participant: “I had to make a decision and go to a place where I‘m sure my family will be better off” (GT07).

### 3.2. Feeling Part of a Community

The second theme emphasizes the importance to respondents of being part of a group. The desire for ties with others, relationality, and the need to belong to a social and cultural group is an expression of the relational dimension of man. Significantly, the need to become an accepted part of society in terms of reciprocity, equal exchanges and proportional relationships is particularly felt by migrants as individuals who, by definition, unlike natives, move, sometimes against their will, to new areas where they will be called to live. The inclusion of migrants is not declined in a one-way assimilationist perspective. Still, it presupposes welcoming diversity, reformulating one’s ideas according to it, and rethinking the other as the bearer of a cultural system that can be considered an added value to the community. *“We hold on to our roots, and we feel good when these are recognized, accepted as a value even for this country that welcomes us”* (VE18).

Interviews show how the level of integration experienced has the power to promote in the other a sense of identity belonging, directly affecting one’s well-being: *“Here I feel better than I did in my country, in the sense that they treat me as I treat them, I don’t feel like a foreigner, they treat me well”* (LO12).

The need to feel part of a community consists of three specific sub-themes.

#### 3.2.1. Reception, Support, and Inclusion

The first sub-theme concerns the need for the migrant to be welcomed, supported, and included in the host country. This sub-theme emphasizes the need for the migrant to receive an act of openness; this means recognizing the other, making them part of something of their own, building mutually enriching relationships, or putting the other in a position to ask for help and be helped: *“Here we live like a family, so they are the ones who take care of everything (...) if you need something or anything else you have to go to them and tell them, and they will try to help you (...) they are like a family (...) there is a cooperation with the other person”* (IR09).

Living in a welcoming family dimension fosters bonding with others and influences one’s perception of oneself: “I feel like I was born here, I feel like this, even though I just arrived, I feel like I‘ve been here for a long time because of the bond that has been created with people” (XC16).

#### 3.2.2. Language Learning as a Tool for Integration

The second sub-theme focuses on language learning as a critical tool for promoting migrants’ well-being. Interviews reveal how language learning is seen as a form of enrichment of one’s identity and an indispensable tool for social inclusion in terms of job opportunities, use of services, and socialization: *“I have to study the language more, which is an important part of integration and being able to be comfortable here (...) the first thing you have to do in the country where you live is to learn the language”* (GT07).

“My future needs to study the Italian language. Without that, I can’t do anything (...) well, starting with learning the language of this country”

(KP11).

The process of adapting to the new communicative environment can start from the possession of a standard language code, an essential requirement for establishing a dialogue with the other and expressing one’s needs and intentions, as well as a means of sharing the experience, which can have positive implications: *“By learning the language we were also able to explain why we are the way we are or why we have the veil and now they have accepted it, or when we are sick and why”* (EV05).

#### 3.2.3. Maintenance of Cultural Identity

The third sub-theme concerns the need to maintain one’s ethnic-cultural identity: acquiring one’s identity occurs through an interactive process constituted by a phase of assimilation and one of differentiation from the other. Therefore, the need for integration and cohesion is accompanied by the need for diversification, that is, the need for identity reappropriation of one’s roots: *“We are integrated, yes, but something is missing, for example, our tradition, and sometimes I feel awful even physically because of that. I would like to find a way to live my culture here too”* (FU06).

The need for migrants to keep alive that identity heritage transmitted by inheritance can only be met if, on the other hand, there is an attitude of recognition and respect for cultural differences that paves the way toward a climate conducive to freedom of expression, and in which the individual can become the authentic spokesperson for a specific system of practices and beliefs related to the culture to which they belong: *“For example, in Libya, we had some prayers to recite, but they were heard outside, and we could not recite them, here in Camini we can. We are free. Or when there is a religious occasion, we can celebrate it freely, a beautiful normal life”* (ZA14).

The practices concern the field of spiritual health, but also traditions linked to well-being and self-care, as stated by this young migrant:

“for example, we use a particular oil to treat children’s wounds, and it is nice to know that the others here understand that it is important for us, more than medicines”

(VE18).

### 3.3. Stability and Security

The third theme generated concerns about the respondents’ need for stability and security. Health, as revealed in the interviews, is linked to the search for better living conditions, in terms of stability, understood as lasting inclusion in the new society of arrival, and security, understood as the need to have a secure base, an anchor of primary importance to feel comfortable. Feeling safe, protected and away from danger is a fundamental need related to individual and group well-being, as well as being a fundamental prerequisite for the realization of needs related to one’s self-realization: *“Feeling safe, I missed so much in my country, where I was always in danger. Now being able to leave home without fear, feeling protected is something that gives me so much well-being”* (VE18). *“I am not well until I feel autonomous, integrated into the country, able to provide for my own life and respond to my children’s wishes”* (CX03).

Two specific sub-themes generate the theme of stability and security.

#### 3.3.1. Independence

The first relates to independence: the interviews reveal the need to achieve individual well-being to gain positive autonomy: *“Being healthy means being autonomous and not needing anyone”* (IR09).

The main aspirations and desires expressed by respondents regarding their future concern the need to learn a trade to acquire skills and abilities helpful in entering the world of work. It is also perceived as essential to maintaining a stable job, through which one can realize one’s need for economic independence, as a source of health: *“The first thing I need is work and being able to support myself economically. Without this, there is no health”* (KP11)

The need for economic independence is often necessary to be able to help one’s family of origin, and it also translates into the need for respondents to access a durable housing solution, which is essential for social inclusion and well-being: *“I want to be able to do something for myself and to support my family. (...) Work to take care of their needs and everything else, and have a home where I can feel good”* (UF19).

#### 3.3.2. The Support of Services

The second sub-theme highlights the importance of access to local services for the well-being of migrants. From the words of the interviewees emerges the vital role that accessible and inclusive services play in facilitating integration and therefore health. Specifically, migrants express the need for support from various community services, such as healthcare, public transport, and childcare. They highlight the importance of these services in creating an enabling environment for them to thrive. One participant expressed this sentiment by saying the following: *“I hope there are more and better services, medical visits, health services that welcome us without discriminating against us and a place where our children can be welcomed, schools, sports” (WD17).*

## 4. Discussion

The present study aimed to explore the perceived health needs of refugees and ASs in Italy. Analysis of the interviews generated three main themes: (1) centrality of family to feel healthy; (2) feeling part of a community; (3) stability and security.

Refugees and ASs converge their health experience around one word: family. Although complex and sometimes compromised by a journey of separations, homesickness, obstacles, and reunifications, family relationships play a decisive role in an individual’s life. Indeed, emigrating means leaving an identity space and a physical one, the unbreakable bond between family members is bound to endure even after leaving the country of origin. Transnational families experience distress resulting from the family separation that can undermine bio–psychosocial integrity and often could be correlated with physical and depressive symptoms [32]. Conversely, living in a family context reduces the risks of falling into deviance [33]. Refugees and AS, as confirmed by our study, direct all the efforts related to adaptation in the new living context to promote their family’s well-being. Our findings also reveal the central role of parenting in the lives of migrants as an issue closely related to health. According to the literature [34], in the post-migratory period, supportive relationships in the family reorganize and become more oriented toward child support, which is undoubtedly the priority element to be activated for. Women, especially mothers, feel a desire to change their lives with the hope that their children do not experience the same sense of instability [35].

The second theme generated, called “Feeling part of a community”, focuses on refugees’ desire to experience a sense of belonging to the community of the host country. They need to have a social life mediated by a feeling of identity, as an expression of membership in the community in which they can feel a part. The innate and fundamental tendency for our species to have interpersonal ties generates well-being; on the contrary, its deprivation, and therefore the lack of interactions, can have negative effects on health [36]. As observed in our study, the need for belonging is expressed by the desire to feel included, welcomed, and supported. Inclusion refers to a complex phenomenon of socio-economic incorporation into the host society and socio-cultural adaptation [37]. Inclusion and welcomeness are two strongly interrelated elements, as each finds its limit in the successful implementation of the other. Finally, social support, such as the possibility of receiving availability, protection, and care from the network of interpersonal relationships, represents the glue in the relationship between the feeling of belonging and individual well-being. Social support is a protective factor for health as it generates positive experiences and reduces stress due to cultural changes experienced by refugees and ASs [38].

The interviewees identify language learning as the primary tool for integration into the target community. Although language integration is not necessarily a guarantee of full integration, acquiring skills in the majority language could undoubtedly facilitate it [39]. The expression in the refugees’ and AS’ positive adaptation process is found in the feeling of belonging to the group of the host country, which represents a crucial factor for psychosocial and economic well-being [40]. Lack of language skills, on the contrary, could be a barrier to the communication of emotional states. Leading to self-isolation and alienation could be significantly associated with a higher prevalence or severity of psychiatric symptoms and mental disorders [41].

Being part of a community is essential for developing and maintaining well-being. Conversely, the perception of cultural discrimination can lead to lower well-being, particularly feelings of guilt, powerlessness, and the individual’s lack of participation in the social and health network [42]. On the other hand, a sense of community and acceptance of cultural uniqueness could protect against the perception of discrimination and its consequences [43]. The real challenge, then, of a system based on reception, is to maintain the identity of the migrant, recognizing cultural roots as valuable elements on which to base interventions of caregiving and treatment. According to Betancourt and Green [44], cultural competence in health care involves: understanding the importance of cultural influences on people’s health beliefs and behaviors; considering how these factors interact at multiple levels of the health care delivery system; and, finally, designing interventions that take these issues into account to ensure quality care. Providing adequate training in intercultural competence is indeed the strategic lever, as Leininger [15] states, for transforming the “stranger” into a person who feels part of a community they recognize as their own and emphasizing the vital role of culturally competent nurses in leading the paradigm shift in reception and caretaking.

“Stability and security” is the third theme identified. For interviewees, the need for security and stability is embodied in the opportunity of gaining or rebuilding complete independence, as a form of labor and housing autonomy. Many studies highlighted how access to the labor market and having stable and safe housing could be two essential aspects for refugees’ and AS’ health [45,46,47]. There is a significant association between refugees’ and AS’ mental health and work quality [48]. Private housing solutions correlate with lower levels of psychological distress and higher levels of life satisfaction than living in shelter environments [49]. In addition, refugees and ASs often face significant barriers to accessing services due to bureaucratic, administrative, and organizational factors [3,50,51]. Studies also highlight the difficulty of designing services focused on the refugees’ and AS’ health needs and aimed at guaranteeing equity and adequate care, both at the moment of first reception and in the long term [3,51].

The present study provides numerous implications for clinical practice. Firstly, it confirms that health is a construct composed of interconnected bio-physiological, psychological, and social dimensions that require an overview rather than an analytical approach focused on the individual domains that comprise it [52]. Second, it provides the key to understanding the priorities of refugees’ and AS’ health needs, which is helpful in socio-health services in their efforts to welcome and care for these populations. Providing services should not be interpreted as a mere act of health and social support but as the possibility, together with the provision of means of subsidy, of promoting autonomy and satisfying the need to feel valid and recognized; pro-sociality could reduce the effects of the social fragility experienced by migrants, promote social integration, and reduce inequalities. Moreover, forced migrants, including refugees and asylum seekers, are initially healthy but often experience deteriorated health once they settle in host countries, experiencing obstacles such as limited access to health care, language, social challenges, difficulties in access to health care, language problems, social challenges, acculturation difficulties, and family issues. These issues underscore the importance of a transculturally trained nurse, who by serving as a bridge between professionals and being trained to take care of the individual and the community holistically, plays a key role in the well-being of this vulnerable population. Starting from the themes emerged from the analysis, many intervention insights arise for the nursing profession. An essential figure to respond to the needs identified through the conducted analyses is the family and community nurse specialized in transcultural nursing. Given that this is an increasingly important reality, implementing interventions aimed at taking care of and providing support for the family unit, making the migrant feel part of a community, and addressing the need to make the migrant feel stable and secure in the new society is urgent.

However, this study also has several limitations. First, it was conducted in a single European country. For this reason, our findings could not be generalizable to other countries, which could have different health systems or different transcultural approaches. Second, the different cultural backgrounds of the participants may have affected the attribution of meaning to the phenomenon explored. In addition, interviewing migrants who were readily available and selected by the host project coordinators may have influenced the participants’ responses and orientation to the phenomena under study. In terms of data collection, interviews were conducted in Italian by the researchers. We acknowledge the invaluable contribution of the cultural mediators from each ethnic group, as this approach proved to be the most effective method for gathering data. However, it is important to recognize that the mediation process may have influenced the depth of communication during the interviews.

## 5. Conclusions

In conclusion, our findings offer valuable insights for healthcare providers operating in transcultural settings. The study underscores the interconnectedness of bio-psychosocial dimensions in refugee health, emphasizing the importance of holistic and culturally sensitive approaches in healthcare delivery. Despite migration challenges, family remains a cornerstone for adaptation and maintaining perceived well-being. Therefore, investing in family support programs and recognizing the role of parents in fostering stability and hope for future generations is crucial. Integration into the host community also emerges as a pivotal factor for well-being. Efforts to promote socioeconomic integration and cultural competence among healthcare workers are thus essential to facilitate transition and reduce isolation. Addressing bureaucratic barriers and designing tailored services prioritizing autonomy and recognition can mitigate challenges faced during settlement phases.

Transculturally trained nurses play a vital role in delivering holistic care and bridging cultural gaps within healthcare systems. By addressing identified needs and challenges, healthcare providers can contribute to promoting resilience, social integration, and well-being among vulnerable migrant populations.

## Figures and Tables

**Table 1:** Sociodemographic variables.

I.D.	GEND.	AGE	Place of Birth	Marital Status	N. Children	Arrival in Italy	ED. Level Country of Origin	ED. Level in Italy	JOB Occupation Country of Origin	JOB Occupation in Italy	Training Courses in Italy
AZ01	F	30	Nigeria	Unmarried	0	2011	Middle School	No	Hairdresser	Hotel	Italian Language
BY02	F	28	Nigeria	Unmarried	2	2016	Middle School	No	Dressmaker	Bag-Labor (Weaving)	Italian Language
CX03	M	41	Morocco	Married	3	2017	High School	Middle School	Security	Bag-Work (Electrician)	Italian Language
DW04	M	18	Syria	Unmarried	0	2016	Middle School	Middle School	No	Ceramist	Ceramist
EV05	F	21	Syria	Unmarried	0	2016	Middle School	High School	E-Commerce	Language Mediator	Italian Language
FU06	F	35	Syria	Married	5	2015	Elementary School	No	No	No	No
GT07	M	33	Morocco	Married	2	2020	Middle School	No	Butcher	Tailor	Italian Language
HS08	F	23	Morocco	Unmarried	0	2021	Middle School	No	No	No	Italian Language
IR09	M	23	Syria	Unmarried	0	2018	High School	High School	Student	No	Italian Language
JQ10	F	24	Somalia	Divorced	1	2018	No	No	No	No	Italian Language
KP11	M	25	Somalia	Divorced	1	2021	No	No	No	No	Italian Language
LO12	F	39	Libya	Married	4	2020	Bechelor Degree	No	No	Tailor	Italian Language
MN13	F	44	Pakistan	Married	4	2014	High School	Middle School	No	No	Italian Language
ZA14	M	47	Pakistan	Married	4	2014	High School	No	Goldsmith	Tailor	Italian Language
YB15	M	24	Bangaldesh	Unmarried	0	2021	No	No	Clerk, Gardener	No	Italian Language
XC16	M	35	Senegal	Married	5	2014	Middle School	Middle School	Farmer	Masoner	Italian Language
WD17	M	28	Lebanon	Unmarried	0	2020	Middle School	No	Employee	Tailor	Italian Language
VE18	F	29	Morocco	Married	2	2020	No	No	No	No	Italian Language, Tailor
UF19	F	30	Nigeria	Married	3	2015	Middle School	Middle School	Telephones Seller	Bag-Labor	Italian Language

**Table 2:** Summar of themes and sub-themes.

Main Theme	Sub-Theme
CENTRALITY OF THE FAMILY TO FEEL HEALTHY	▪ The symbolic role of parental function ▪ The reunification of the family unit ▪ Individual well-being depends on the well-being of the family
FEELING PART OF A COMMUNITY	▪ Reception, support and inclusion ▪ Language learning as a tool for integration ▪ Maintenance of cultural identity
STABILITY AND SECURITY	▪ Independence ▪ The support of services

## Data Availability

The data presented in this study are available from the corresponding author upon request.

## Public Involvement Statement

The authors declare no public involvement in any aspect of this research.

## Guidelines and Standards Statement

This manuscript was written following the recommendations of the COREQ guide for qualitative research reporting (Tong, 2007).
